# *GhCAX3* Gene, a Novel Ca^2+^/H^+^ Exchanger from Cotton, Confers Regulation of Cold Response and ABA Induced Signal Transduction

**DOI:** 10.1371/journal.pone.0066303

**Published:** 2013-06-11

**Authors:** Lian Xu, Kashif Rafiq Zahid, Liangrong He, Wenwen Zhang, Xin He, Xianlong Zhang, Xiyan Yang, Longfu Zhu

**Affiliations:** 1 National Key Laboratory of Crop Genetic Improvement, Huazhong Agricultural University, Wuhan, Hubei, P. R. China; 2 College of Plant Science, Tarim University, Alaer, Xinjiang, P. R. China; New Mexico State University, United States of America

## Abstract

As a second messenger, Ca^2+^ plays a major role in cold induced transduction via stimulus-specific increases in [Ca^2+^]_cyt_, which is called calcium signature. During this process, CAXs (Ca^2+^/H^+^ exchangers) play critical role. For the first time, a putative Ca^2+^/H^+^ exchanger *GhCAX3* gene from upland cotton (*Gossypium hirsutum* cv. ‘YZ-1′) was isolated and characterized. It was highly expressed in all tissues of cotton except roots and fibers. This gene may act as a regulator in cotton’s response to abiotic stresses as it could be up-regulated by Ca^2+^, NaCl, ABA and cold stress. Similar to other CAXs, it was proved that GhCAX3 also had Ca^2+^ transport activity and the N-terminal regulatory region (NRR) through yeast complementation assay. Over-expression of *GhCAX3* in tobacco showed less sensitivity to ABA during seed germination and seedling stages, and the phenotypic difference between wild type (WT) and transgenic plants was more significant when the NRR was truncated. Furthermore, GhCAX3 conferred cold tolerance in yeast as well as in tobacco seedlings based on physiological and molecular studies. However, transgenic plant seeds showed more sensitivity to cold stress compared to WT during seed germination, especially when expressed in N-terminal truncated version. Finally, the extent of sensitivity in transgenic lines was more severe than that in WT line under sodium tungstate treatment (an ABA repressor), indicating that ABA could alleviate cold sensitivity of GhCAX3 seeds, especially in short of its NRR. Meanwhile, we also found that overexpression of *GhCAX3* could enhance some cold and ABA responsive marker genes. Taken together, these results suggested that GhCAX3 plays important roles in the cross-talk of ABA and cold signal transduction, and compared to full-length of *GhCAX3*, the absence of NRR could enhance the tolerance or sensitivity to cold stress, depending on seedling’s developmental stages.

## Introduction

Plant needs to adapt to unfavorable environmental conditions that include biotic and abiotic stresses, like drought, salinity and cold [1], [2]. Cold is one of the major threats to crop plants and could be categorized as chilling stress (0°C to 10°C) and freezing stress (<0°C) [3]. Each plant species has different temperature ranges for its proper growth and development. Temperature conditions, which are suitable for the growth of one plant, may be stressful for another plant. It has been observed that plants in hot climate show symptoms of injury upon exposure to low non-freezing temperatures. Cold stress during the reproductive stage of the plants delays heading and results in pollen sterility, which is regarded as major factor for reduction of yield of crops [4]. Many physiological and biochemical cell functions, like cell membrane structure and lipid composition [5], cellular leakage of electrolytes [6] and protoplasmic streaming and redistribution of intracellular calcium ions [7] have been correlated with visible symptoms, like wilting, chlorosis, or necrosis when plants are exposed to low temperature.

After exposure of plants to environmental stresses, a signal is generated, collectively known as signal transduction, which always starts with the perception of the abiotic stress. As a second messenger, calcium plays an important role in the signal transduction involved in various abiotic stresses. It has already known that there is a transient increase in [Ca^2+^]_cyt_ in plant cells during stress, however, the pattern or temporal dynamic of this [Ca^2+^]_cyt_ elevations, named as oscillations, are specifically stimulated, and this stimulus-specific Ca^2+^ signals are known as a “Ca^2+^ signature” [8]. The dynamic feature of the oscillation can be viewed as the result of [Ca^2+^]_cyt_ influx and efflux events including CAXs (Ca^2+^/H^+^ exchangers) in the plasma membranes and endomembranes that mediate transport of [Ca^2+^]_cyt_ in or out of vacuole/plasma membrane [9].

CAXs were firstly identified by suppression of Ca^2+^ sensitivity in yeast, diverse functions and molecular properties for CAXs in plants were then identified [10]. Initially, Plant cation/H^+^ exchangers were cloned by means of condensed N-terminal proteins to function in the yeast mutants defective in vacuolar Ca^2+^ transport [10], [11]. This N-terminal auto-inhibitory domain exists in a variety of plant CAXs and decreases their activity in both yeast and plants [12]. This auto-inhibition is caused by the N-terminus physically interacting with a neighbouring N-terminal region, and also, suppression of CAXs activity could be abolished via interaction between the N-terminus and some smaller proteins [13]. To date, some CAXs have been identified in physiological roles, biochemistry and molecular biology from *Arabidopsis*, soybean and rice [14], [15], [16] whereas there’s no report on CAX from cotton.

It has been proved that CAX transporters play important roles in salt and cold stresses by mediating cation transport or abiotic stress induced signal transductions, yet the regulation of CAX’s expression was still unclear. As a Ca^2+^/H^+^ transporter, sAtCAX1 (N-terminal truncated version) was reported to improve Ca^2+^ accumulation and Ca^2+^-related stress (salt and cold) sensitivity in tobacco plants due to its constitutive activity. However, GmCAX1 and CrCAX1 could confer salt tolerance in *Arabidopsis* due to the Na^+^/H^+^ transport activity [15], [17]. Furthermore, *cax3* mutant of *Arabidopsis* was found more sensitive to salt that resulting in decreased plasma membrane H^+^-ATPase activity, which indicated that AtCAX3 might be involved in salt induced signal transduction although the mechanism was not clear. It was also observed that *Arabidopsis cax1* showed more tolerance under freezing temperature after cold accumulation. However, there was no difference in their chilling and constitutive freezing tolerance as compared to WT, which inferred that AtCAX1 plays a negative role specifically in cold accumulation [18]. The result was consistent with the symptom that *sAtCAX1*-overexpressed tobacco showed more cold sensitivity [19], although the mechanism between these two symptoms was different. The cold tolerance phenotype in *cax1* attributed to CAX1’s participation in Ca^2+^ signaling involved in CBF/DREB1 mediated signaling pathway [18]. Besides this, *sAtCAX1*-overexpressed tobacco and tomato showed Ca^2+^ deficiency symptoms even under normal condition. These result indicate that CAX1 could participate in [Ca^2+^]_cyt_ homeostasis and [Ca^2+^]_cyt_ induced signal transduction [20].

Previously, one EST (DT458383) of cotton responsive to multiple abiotic stimuli was isolated based on data-mining [21]. Here, we obtained the full length of the gene, which putatively encodes a Ca^2+^/H^+^ exchanger, and proved the existences of its N-terminal regulatory region (NRR) and Ca^2+^ transport function through yeast complementation assay. Furthermore, the responses of transgenic plants to ABA and cold suggested a novel regulatory role of GhCAX3 in ABA and cold signal pathways.

## Materials and Methods

### Plant Cultivation and Treatment

Seeds of ‘YZ1’ (*G. hirsutum* L.) were soaked in wet gauze until complete germination. Fully germinated seeds were transferred to pots at 28°C under controlled conditions (16 h light/8 h dark photoperiod). After the emergence of leaves, cotton seedlings were incubated in solution containing 200 mM CaCl_2_, 400 mM NaCl, 15% (W/V) PEG and 100 µM ABA. For cold stress treatments, the cotton seedlings were transferred to growth chambers with same photoperiod at 4°C for 24 h. Samples were collected at 0, 1, 4, 8, 24 and 48 hours later under Ca^2+^ treatment and 0, 3, 6, 12 hours after salt, PEG and cold treatment. During ABA treatment, samples were collected at 0, 0.5, 1 and 4 hours. Seedlings under normal growth conditions were used as control. All the samples were frozen in liquid nitrogen immediately after collection and stored at −80°C.

### Identification of Full-length cDNA and qRT-PCR Analysis

Total RNA was extracted from cotton leaves and roots after exposure to various environmental cues, according to the method of Zhu et al. [22]. Reverse transcribed cDNAs were synthesized by using 3 µg of total RNA with the Script III reverse transcriptase (Invitrogen, Carlsbad, USA). Rapid-amplification of cDNA ends-PCR (RACE-PCR) were used to amplify the full-length of the Ca^2+^/H^+^ exchanger (CAX) gene from cotton. The PCR product was purified, cloned into the pGEM-T Easy vector (Promega, USA), and transformed into competent *E. coli* cells for sequencing. The amino acid sequence alignment and phylogenic analysis of GhCAX3 protein and its homologues was conducted using Clustal X software. Hydropathy profile of GhCAX3 was predicted according to Anthe analysis, and transmembrane domain analysis was constructed by using TMMOD (http://molbiol-tools). qRT-PCR (quantitative real-time PCR) analysis of *GhCAX3* was performed with gene specific primers RCAX-F and RCAX-R (Table S1), by using the ABI Prism 7000 (Applied Biosystems, Foster City, USA).

### Vector Construction, Yeast Transformation and Characterization by Northern Blot

The Ca^2+^ sensitive yeast mutant strain K667 (*cnb1*::*LEU2 pmc1*::*TRP1 vcx1*) was used as a host to test the function of *GhCAX3*. To confirm the existing of N-terminal auto-inhibitory domain in *GhCAX3*, three primers, YCAX-F, sYCAX-F and YCAX-R (Table S1) were used to amplify the full-length *GhCAX3* (1–448 aa) and N-terminal truncation version *sGhCAX3* (31–448 aa). Both the *GhCAX3* and *sGhCAX3* were ligated at *XbaI* and *SacI* sites of piHGpd shuttle vector under the control of Gpd promoter and transformed into the K667 using lithium acetate method. Positive clones were screened and selected on synthetic complete minus His (SC-His) media. For negative control, the vector piHGpd was transformed into K667 strain and the same vector was transformed into the corresponding wild type strain W303-1A as a positive control.

Total RNA was extracted from yeast following the method of Wang et al. [23], fractionated in a 1.0% agarose gel containing formaldehyde and blotted onto a Hybond-Nylon membrane (Millipore, USA). For probe preparation, two specific primers PCAX-F and PCAX-R (Table S1) were designed based on *GhCAX3* cDNA sequence to amplify the specific sequence, and northern blot hybridizations were performed according to protocols of Tu et al. [24].

### Ca^2+^ Transport Assay and Stress Experiments in Yeast

Yeast K667 strains were transformed with *GhCAX3*, *sGhCAX3* and empty vector, and the wild type strain W303-1A transformed with empty vector were used for Ca^2+^ transport assay and stress experiment. When the strains were grown to OD_600_ of 1.0 in liquid SC-H media [25], 100 µL of various dilutions were replicated in YPD solid medium containing 0–200 mM CaCl_2_ for 3 days. Ca^2+^ tolerance assay was also performed in liquid YPD media, 100 µL of strains with OD_600_ of 1.0 were inoculated into YPD liquid medium supplied with 0–200 mM CaCl_2_ and allowed to grow for 20 h. For salt stress experiment, 100 µL of strains were spotted and grown in YPAD medium containing 0.75 M NaCl and 0.25 M LiCl for 3 days. For cold tolerance analysis, same strains were replicated in YPAD medium at 4°C for 3 weeks before transferred for recovery at 30°C for 2 days. All the strains were grown at 30°C except cold stress.

### Construction of Plant Expression Cassettes and Tobacco Transformation

For the full-length amplification of *GhCAX3* and N-terminal truncated *sGhCAX3*, attB1 and attB2 were linked to gene-specific primers TCAXF, TCAXR, sTCAXF and sTCAX3R (Table S1). BP reaction of the PCR products with pDONR221 was carried out and then *GhCAX3* and *sGhCAX3* sequences were inserted into pGWB417 and pGWB418 separately from pDONR221 clones by LR reaction [26]. The pGWB417-*GhCAX3* and pGWB418-*sGhCAX3* constructs were transferred into *Agrobacterium tumefaciens* EHA105 by electroporation. Transformation in tobacco (*Nicotiana benthamiana*) was performed by leaf disc method [27]. Transgenic plants were detected by PCR analysis using primers PCAX-F and PCAX-R (Table S1), and single copy lines were screened on 1/2 MS medium containing 35 µg/ml of kanamycin.

### Germination and Stress Experiments in Tobacco

For ABA and salt stress to germination assays, 150 seeds each from transgenic tobacco and wild type line were sown on 1/2 MS medium supplied with 150 mM NaCl and 5 µM ABA at room temperature. For cold stress treatment, seeds were sown at different temperature from 10°C–24°C (10°C, 15°C, 18°C, 21°C and 24°C). Germination experiment was also performed with supplementation of sodium tungstate at a concentration of 0, 0.01, 0.1 and 1 mM on 1/2 MS medium under 24°C and 18°C. During ABA and NaCl treatment, photographs were taken after 14 days, whereas under sodium tungstate and cold stress treatment, photographs were taken after 12 days. Each experiment was repeated 3 times and statistical analysis of germination rate was performed after three weeks. For stress experiment in tobacco, seedlings of transgenic and WT lines were grown on 1/2 MS medium for 12 days, and then transferred into pots at 24°C under controlled conditions. Six-week old plants were used for both the ABA and cold stress treatments. For cold stress, plants were exposed to 0°C (±1°C) for 48 h and then 7 days for recovery at 24°C. For ABA treatment, plants were sprayed by 100 µM ABA solution.

### Stress-responsive Gene Expression Analysis by RT-PCR

Total RNA was extracted with Trizol Regent Kit (Invitrogen, USA). 3 µg of total RNA was reverse-transcribed to cDNA. Total 20-µl-reaction volume was used for RT-PCR analysis to examine expression of this gene. The PCR reaction mixture was subjected to 94°C denaturation for 5 min, then 30 cycles of 94°C denaturation for 30 sec, 58°C annealing for 30 sec and 72°C extension for 1 min, plus a final extension at 72°C for 5 min. Genes involved in ABA or cold stress signal transduction, including *NbDIN*, *NbERD10C*, *NbRab11D* and *NbSOD*, were selected and the primer sequences were used as previously described [28].

### Measurements of Morphological and Physiological Parameters

Transgenic and wild type tobacco seedlings were first grown on 1/2 MS medium for one week, and then transferred to 1/2 MS medium with or without different concentration of ABA for 2 weeks, and the main root length was measured. Relative electrolyte leakage (REL) was measured according to the method of Yang et al. [29]. Catalase (CAT) activity and Peroxidase (POD) activity were measured according to method described by Corbisier et al. [30] and Polle et al. [31].

## Results

### Identification and Expression Analysis Of *Ghcax3* Under Diverse Environmental Stresses

In our earlier effort of data mining for abiotic stress responsive genes in cotton [21], one gene with an EST identification of DT458383 that might participate in abiotic stresses was selected for further study. The full-length cDNA clone of 1824 bp, containing an ORF of 1347 nucleotides, was obtained through RACE method, which encodes a peptide of 448 amino acid residues with a predicted molecular mass of 49 KDa (Figure S1A). The protein was named as GhCAX3 because the alignment results showed that it was highly homologous to AtCAX3, with 74% identity and 84% similarity, respectively. Phylogenic analysis showed that GhCAX3 belongs to type IA CAX and was also very close to AtCAX1 (Figure S1B). Similar with some other CAXs, GhCAX3 contains a NRR and one 9-amino acid domain (CaD), as predicted by hydropathy and amino acids analysis. Besides this, the NRRs are more conserved among type IA CAXs, whereas CAXs of type IB (AtCAX2, oSCAX3 and oSCAX3) possess various versions of NRR. None of CaD domains are identical among CAXs (Figure S1A, S1C). Eleven transmembrane domains conserved among CAXs were identified by hydropathy and transmembrane domain analysis (Figure S1C-D).

**Figure 1:**
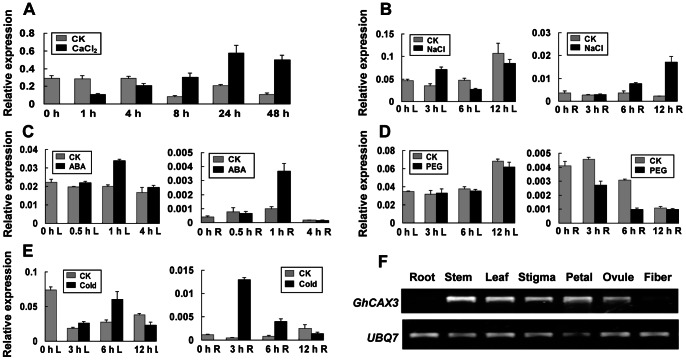
Expression pattern analysis of *GhCAX3* in cotton responsive to various treatments. Cotton seedlings were treated with 200 mM CaCl_2_ (**A**), 400 mM NaCl (**B**), 100 µM ABA (**C**) and 15% (W/V) PEG (**D**). For cold stress measurement, seedlings were exposed to 4°C (**E**). Total RNA was isolated from leaf (L) tissues at indicated time points for *GhCAX3* expression assay under Ca^2+^ treatment. Under salt, ABA and PEG treatment, total RNA from leaf (L) and root (R) tissues was isolated respectively at indicated time points. Expression analysis was tested by qRT-PCR. Grey and black column represent relative expression level of *GhCAX3* with respect to *UBQ7* under normal condition (CK) and various cues, respectively. (**F**) *GhCAX3* transcripts in various tissues of cotton were detected by RT-PCR. Root, stem and leaf samples were collected from 3-week old seedlings, the other tissues were from mature cotton plants.

*GhCAX3* was responsive to Ca^2+^ treatment and the expression level was declined at 1 and 4 h, while a markedly sustained increase was detected 8 h after Ca^2+^ treatment (Figure 1A). The expression pattern showed that the transcripts of *GhCAX3* were increased in response to NaCl, ABA and cold in cotton (Figure 1B-C, 1E). It could be induced dramatically in roots by cold and ABA in very short time. The expression was decreased in root but no more change was observed in leaf tissues during PEG treatment (Figure 1D). The transcript level of *GhCAX3* was studied in different tissues in cotton by RT-PCR (Figure1F). It appears that GhCAX3 was highly expressed in leaf, stem, stigma, petal and ovule, in comparison with the low levels seen in root and fiber.

### Ca^2+^ Transport and Stress Tolerance Assay of *GhCAX3* in Yeast

**Figure 2:**
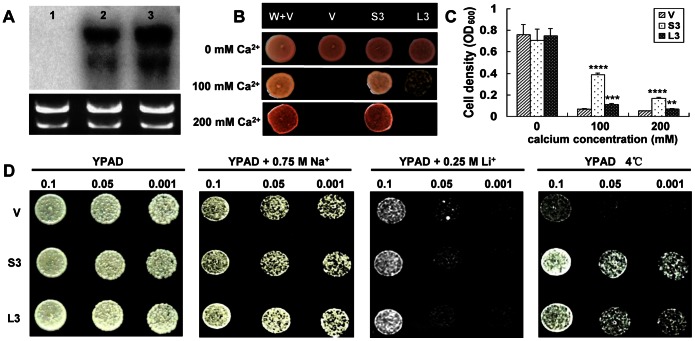
Characterization of *GhCAX3* and *sGhCAX3* in yeast. (**A**) Expression analysis of *GhCAX3* and *sGhCAX3* in K667 by northern blot. The blot was hybridized with ^32^P-labelled GhCAX3 cDNA probe. 1, K667 yeast strain with empty vector; 2, K667 yeast strain with sGhCAX3; 3, K667 yeast strain with GhCAX3. Ethidium bro-mide-stained total RNA is shown at the bottom. (**B–C**) Analysis of Ca^2+^ sensitivity of the yeast with *sGhCAX3* and *GhCAX3*. Recombinant cells were spotted onto YPD solid (**B**) and liquid (**C**) medium containing 0–200 mM Ca^2+^. Yeast cells were allowed to grow for 3 days at 30°C before photograph was taken. W+V: wild type W303-1A with empty vector; V: K667 with empty vector; S3: sGhCAX3 expressed in K667 cells, L3: GhCAX3 expressed in K667 cells. (**D**) Effect of sGhCAX3 and GhCAX3 in yeast under different abiotic stresses. Liquid cultures of K667 cells expressing GhCAX3, sGhCAX3 and empty vector were serially diluted into 0.1, 0.05 and 0.001 times of original density, spotted on YPAD plate with 0.75 M NaCl and 0.25 M LiCl and grown at 30°C for 3days. For cold stress, yeast was grown on YPAD medium at 4°C for 3 weeks before transferred to normal condition for two-day recovery. **, *** and **** indicate significant differences relative to the control at P<0.01, P<0.001 and P<0.0001, respectively.

Previous study showed that most CAXs had Ca^2+^/H^+^ transport function and an auto-inhibitory domain in their N-terminal. We were interested in testing whether GhCAX3 had Ca^2+^/H^+^ transport activity and could be auto-inhibited by its N-terminal. Thus both the full-length of *GhCAX3* and the corresponding N-terminal truncated version *sGhCAX3* were inserted into yeast expression vector piHGpd. These two constructs were transferred into a yeast mutant strain k667 to test the Ca^2+^ transport activity and the role of GhCAX3’s N-terminal. RNA blot analysis revealed two transcript bands for both GhCAX3 and sGhCAX3 recombinant yeast strains, and no transcript band from the vector containing yeast strain (Figure 2A). The wild type strain W303-1A with empty vector (W+V) and the recombinant K667 cells with *sGhCAX3* (S3) grew well in solid YPD medium supplemented with 100 mM and 200 mM Ca^2+^, whereas K667 cells with empty vector (V) failed to grow. The growth of *GhCAX3* strains (L3) was partially suppressed in 100 mM Ca^2+^ containing medium while was completely suppressed when the concentration of Ca^2+^ was raised to 200 mM (Figure 2B). Although both strains with *GhCAX3* and vector were unable to grow in presence of 200 mM Ca^2+^ in solid YPD medium, statistical analysis of cell density showed that there was significant difference between them in liquid YPD medium. *GhCAX3* cells density showed 70% and 39% higher compared with empty vector expressed cells in presence of 100 mM and 200 mM Ca^2+^. Moreover, the cells density of yeast expressing *sGhCAX3* was significantly higher than that of *GhCAX3* and empty vector expressed cells in presence of Ca^2+^ (Figure 2C). No significant difference in growth was observed among these strains in liquid YPD medium under normal condition.

We also characterized the function of GhCAX3 in yeast response to abiotic stresses. Various dilutions of all recombinant K667 cells were spotted on YPAD medium supplied with 0.75 M NaCl and 0.25 M LiCl at 30°C and on YPAD medium at 4°C. There was no noticeable difference under salt stress (NaCl and LiCl), while strains with *GhCAX3* (L3) and *sGhCAX3* (S3) grew much better than that with empty vector (V) after exposure to 4°C (Figure 2D). Meanwhile, slight difference was also observed between *GhCAX3* and *sGhCAX3* expressed cells during cold stress, in which *sGhCAX3* conferred a little more tolerance in yeast strains.

### Overexpression of *GhCAX3* in Tobacco Alleviates ABA Sensitivity

**Figure 3:**
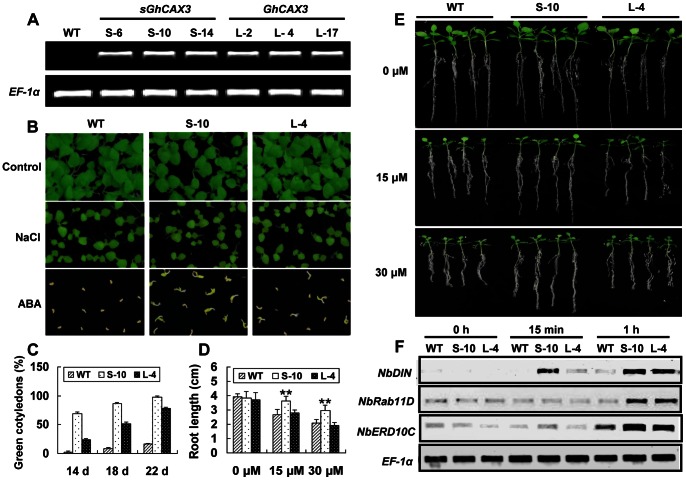
Expression of *GhCAX3* and *sGhCAX3* could impair ABA sensitivity in tobacco plants. (**A**) Expression analysis of *GhCAX3* and *sGhCAX3* in tobacco by RT-PCR. The *EF-1α* gene was used as an internal control. (**B–C**) Comparison of seed germination in WT and transgenic lines under NaCl and ABA treatments. (**B**) Phenotypic analysis of WT and transgenic seeds germinated on 1/2 MS medium for two weeks under normal condition and in presence of 150 mM NaCl as well as 5 µM ABA. (**C**) Calculation of germinating seeds with green cotyledons. Percentage of germinating seeds with green cotyledons between WT and transgenic lines grown on 1/2 MS medium containing 5 µM ABA was recorded up to 22 days. (**D–E**) Statistical (**D**) and phenotypic (**E**) analysis of WT and transgenic lines after two weeks treatment by ABA. S-10, L-4 and WT seeds were germinated on 1/2 MS medium for 7 days before transferred onto 1/2 MS medium with different concentrations of ABA as indicated. Photograph was taken after 2 weeks growth. (**F**) Expression analysis of stress-related genes by RT-PCR. Total RNA was extracted from leaf samples at the indicated time points after ABA treatment. Each column in C and D represents an average of four repeats. ** indicates significant differences relative to the control at P<0.01.

As *GhCAX3* in full-length could improve cold tolerance in yeast as well as its N-terminal truncated form, to further investigate its biological function against abiotic stresses in plants, we inserted *GhCAX3 and sGhCAX3* into tobacco (*Nicotiana benthamiana)* via *Agrobacterium tumefaciens*-mediated method under the control of Cauliflower Mosaic Virus (CaMV) 35S promoter. A total of 41 independent transgenic lines for two constructs were generated and three single copy insertion lines for each construct were selected by Kanamycin resistance selection. Transcripts of GhCAX3 and sGhCAX3 in transgenic tobacco plants were analyzed by RT-PCR. Two overexpressed tobacco lines, L-4 and S-10 with relatively high expression from *GhCAX3* and *sGhCAX3* overexpressed tobacco, respectively, were chosen for further experiments (Figure 3A).

We first examined the transgenic tobacco plants in response to ABA and salt during seed germination and seedling development. All the seeds were germinated on 1/2 MS medium or 1/2 MS medium supplemented with ABA and NaCl, respectively (Figure 3B). Germination rate of transgenic lines and WT was almost the same under normal condition as well as in presence of NaCl, although the germination of all these seeds was delayed under salt treatment (data not shown). However, under 5 µM ABA treatment, germination of WT was severely inhibited, whereas overexpressing *GhCAX3* or *sGhCAX3* in tobacco impaired the sensitivity to ABA as compared to WT (Figure 3B-C). Similar results were found during seedling development responsive to ABA. Under 15 and 30 µM ABA treatment, seedlings of S-10 showed longer root length than that of WT. While no more difference in root length was found between L-4 and WT seedlings (Figure 3D-E). The experiment for salt stress analysis at seedling stage was also performed while no difference was observed between WT and transgenic lines (data not shown). To test whether s*GhCAX3* could alter the expression of ABA responsive genes, two known ABA up-regulated genes *NbRab11D* and *NbDIN* and [32], [33] were examined by RT-PCR. Transcript level of these two genes was higher in transgenic plants, between which *NbDIN* was induced more quickly in S-10 line (Figure 3F).

### Overexpression of *GhCAX3* Confers Cold Sensitivity on Seed Germination and Tolerance on Seedlings, Respectively

**Figure 4:**
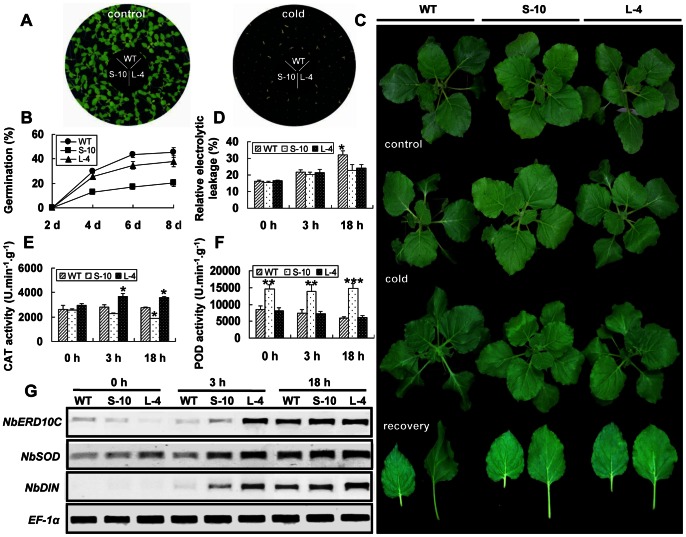
Characterization of *GhCAX3* involved in cold stress response in tobacco. (**A**) Photograph was taken when S-10, L-4 and WT seeds were sown on 1/2 MS medium at normal temperature (24°C) and 18°C for 12 days. (**B**) Evaluation of germination rates in WT and transgenic lines at indicated time points after sowing at 18°C. (**C**) Phenotypic analysis of plants response to cold stress. Six-week old tobacco plants were exposed to 0°C (±1°C) for 2 days and then transferred to 24°C in greenhouse for one-week recovery. (**D–F**) Analysis of relative electrolyte leakages and activities of CAT and POD in WT and transgenic plants after cold treatment. Leaf samples were collected at indicated time points for measurement, data was presented as means from three repeats. (**G**) Expression analysis of stress-responsive genes under cold treatment by RT-PCR.

Temperature is one of the important factors to affect germination of seeds. To characterize the function of *GhCAX3* in response to low temperature during germination, seeds of transgenic lines and wild type were grown on 1/2 MS medium at different temperatures from 10°C to 24°C. The results showed that seed germination of tobacco was very sensitive to temperature. When the temperature was below 15°C, no germination of all seeds could be seen even after 10 days. The rate of germination at 21°C was slower than that at 24°C, while the percentage of germination was similar after 6 days (data not shown). Much more difference in germination was found in the results at 18°C. The germination rate of wild type was significantly higher among the three lines, while the percentage of germination of S-10 seeds was much lower than that of WT and L-4 lines (Figure 4A-B).

To further investigate the role of *GhCAX3* in plant during cold stress, six-week-old seedlings were grown at 0°C (±1°C) for 48 h, and then recovered at normal condition for seven days. In contrast to the results of seed germination under low temperature, seedlings of WT were severely damaged with more wilting leaves under cold stress in 48 h, while S-10 seedlings showed more tolerance to cold stress (Figure 4C). After one-week recovery, transgenic plants resumed normal growth but the leaves of WT retained curled from middle to bottom part of the plant (Figure 4C). Similar results were obtained in three repeated experiments which indicated that overexpression of GhCAX3 in tobacco seedlings could enhance tolerance to cold stress.

As a physiological indicator of membrane damage, relative electrolyte leakage was measured to evaluate the effect of GhCAX3 in physiological response to cold stress. There was no much difference between transgenic and WT lines in electrolyte leakage after 3 h at 0°C, however, obvious difference was seen after 18 h cold treatment. There was more membrane damage happened at low temperature in wild type leaves because of higher electrolyte leakage compared with the transgenic plants. No obvious difference was found in electrolyte leakage between S-10 and L-4 lines, though there was always a little low in S-10 plants (Figure. 4D). We also measured the activity of two antioxidant enzymes in plants under cold stress. At normal condition, no significant difference was observed in CAT activity between WT and transgenic plants, although it was a little higher in L-4 line. During cold treatment, CAT activity was induced in a similar manner in L-4 and WT plants, however, it was significantly lower in WT than that in L-4 line. Besides this, it was very interesting to find that the activity of CAT in S-10 line constantly declined during the cold treatment and was significantly lower than that of WT and L-4 line at 18 h (Figure 4E). Compared with the activity of CAT, the activity of POD in S-10 line was markedly higher than that of L-4 and WT lines even under normal condition, indicating that the activities of antioxidant enzyme either in GhCAX3 or sGhCAX3 lines were enhanced although their inducible pattern was different (Figure 4F).

In addition, the expression level of *NbSOD* and the cold responsive marker gene *NbERD10c* (one of a family genes encoding group 2 LEA proteins) was increased more fast and abundant in transgenic plants as compared to WT line, although the expression level of these two transcripts was a little lower in S-10 line than that in L-4 line at 3 h after stress (Figure 4G).

### Involvement of *GhCAX3* in Regulation between ABA and Cold Signal Transduction

**Figure 5:**
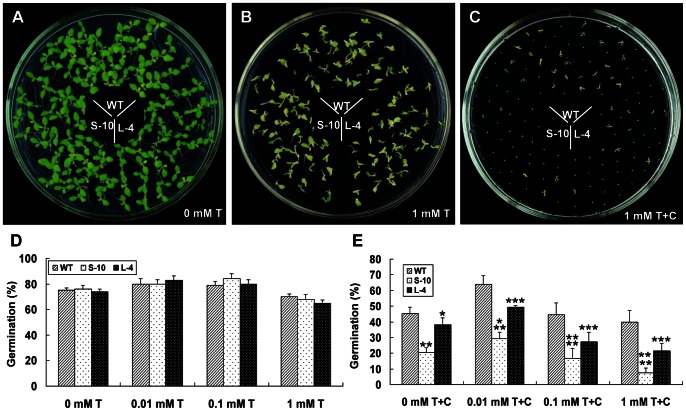
Cross-talk analysis of GhCAX3 involved in ABA and cold stress signal pathway. (**A**) Seed germination of S-10, L-4 and WT grown on 1/2 MS medium under normal condition for 12 days. (**B–C**) Seed germination of S-10, L-4 and WT grown on 1/2 MS medium with 1mM sodium tungstate for 12 days at 24°C and 18°C, respectively. (**D–E**) Statistical assay of germination rate of transgenic and WT lines germinated on 1/2 MS medium with a series of concentration of sodium tungstate at 24°C (**D**) and 18°C (**E**) for 12 days. Mean values were shown from at least four replicates. *, **, *** and **** indicate significant differences relative to the control at P<0.05, P<0.01, P<0.001 and P<0.0001 respectively. T: Medium with sodium tungstate; C: Cold treatment at 18°C.

Although cold-induced signal transduction was thought to be independent from ABA pathway, cross-talk between two signaling pathways may exist [34]. In our study, it showed that *GhCAX3* participated in both the cold and ABA signaling pathways, so we were interested in testing whether the cross-talk was mediated by *GhCAX3*. Seeds of transgenic and WT lines were sterilized and grown on 1/2 MS medium with or without 1 mM sodium tungstate (an ABA biosynthesis inhibitor) at 24°C and 18°C, respectively, and the percentage of germination was calculated at 12 day after sowing. Statistical analysis showed that there was not much difference in the germination rates between transgenic and WT lines at 24°C with sodium tungstate in the medium. However, compared to normal condition, the germination rate was increased a little with the concentration of sodium tungstate at 0.01 mM and 0.1 mM, while high concentration of sodium tungstate at 1 mM could inhibit the germination and delay the development of seedlings (Figure 5A-B, 5D). Great difference was found when the seeds were grown at 18°C with sodium tungstate, the rate of germination of all lines decreased obviously compared with that in the normal condition (Figure 5C, 5E). And among them, the rate of germination of WT in the medium at different content of sodium tungstate was the highest one, and that of S-10 was the lowest one. Meanwhile, rate of germination of all three lines was increased about 20% at 18°C with 0.01 mM sodium tungstate compared with that without sodium tungstate, and decreased at the higher content of sodium tungstate. In comparison with *GhCAX3*, overexpression of *sGhCAX3* in tobacco made the seeds more sensitive to sodium tungstate at 18°C. The germination rate of S-10 tobacco in this condition decreased 53% under 1 mM sodium tunastate treatment while 40% decrease was found in L-4 line (Figure 5E).

We also checked some marker genes involved in ABA or cold stress signal pathway. *NbERD10C*, as a cold-responsive gene, could be induced by ABA in about 1 h, and more abundant transcripts were found in the transgenic lines at the same time (Figure 3F). Meanwhile, *NbERD10C* in S-10 line was induced more quickly in response to ABA (Figure 3F). The ABA responsive gene *NbDIN* was more rapidly and strongly induced by cold stress treatment in transgenic plants. It was induced within 18 h in WT plants, while the transcript level of this gene started to increase within 3 h in transgenic plants and continued to increase for 18 h. Compared with line L-4, the expression of *NbDIN* in S-10 was a little lower (Figure 4G). All these results indicated that *GhCAX3* could participate in the interaction between ABA and cold signal transduction.

## Discussion

Based on data mining method, we have isolated a large number of putative abiotic stress responsive genes in cotton [21], but the biological functions of the candidate genes were not confirmed. According to the results, one gene, *GhCAX3*, was obviously up-regulated by cold and ABA in short time especially in roots, indicating that it may be responsive to stress, and this deduction was subsequently validated by the results from cold stress and ABA treatment experiment in yeast and tobacco. Nevertheless, no evidence was found that *GhCAX3* could influence the salinity response in yeast and tobacco plants although its transcript level also significantly increased in roots under salt stress in cotton. Plants cautiously keep [Ca^2+^]_cyt_ homeostasis to prevent Ca^2+^ toxic accumulation and deficiency, and also to fulfill their role during cellular signal transduction under abiotic stresses. CAXs are considered to be important in adjusting these events. As a Ca^2+^/H^+^ exchanger, GhCAX3 may be involved in the up-stream of the signal pathway due to the response in short time after cold and ABA treatments. All these proved the feasibility of this method to screen stress-related genes in large scale in cotton, while the specific role of the candidate genes needs to be demonstrated in experiment.

Transcripts were confirmed by RNA blot analysis when GhCAX3 was characterized in yeast, two bands were observed in both the *sGhCAX3* and *GhCAX3* expressed strains. Similar results were also found on RNA and protein level from some other reports about CAXs, either in full-length or N-terminal truncated form, when expressed in yeast or tomato [20], [35]. And this might be attributed to multiple transcriptional initiation sites in the gene. Previous study has demonstrated the existence of NRRs, which modulates Ca^2+^ transport through N-terminal auto-inhibition, among some plant CAXs [16]. In our research, sGhCAX3 could efficiently suppress the Ca^2+^ sensitive phenotype of K667 strains in the presence of Ca^2+^ while full length of GhCAX3 weakly suppressed the Ca^2+^ hypersensitivity of K667 strains under 100 mM Ca^2+^ treatment. Although GhCAX3 cells failed to grow on YPD medium supplied with 200 mM Ca^2+^, its cell density increased more significantly than that of control in the same condition (Figure 2B-C). All of these results indicated that the NRR was also located at the N terminal of GhCAX3 and could eliminate most of its Ca^2+^ transport activity, and the growth failure of GhCAX3 cells on YPD medium with 200 mM Ca^2+^ might be due to that the activity of full length GhCAX3 was not high enough to cause phenotypic difference between cells with GhCAX3 and the control under high concentration of Ca^2+^ treatment. Compared with other CAXs, like AtCAX1 and CrCAX1, the activity of GhCAX3 was more severely inhibited by its NRR, as full length of the two mentioned proteins could partially recover the growth of K667 cells in the presence of 200 mM Ca^2+^ [17], [36]. As a CAX with high homology to AtCAX3, GhCAX3 showed a different mechanism in N-terminal regulation. Unlike sGhCAX3, sAtCAX3 failed to suppress yeast Ca^2+^ deficiency until extension deletions beyond the N-terminal ∼40-amino acid NRR that previously deduced [37].

ABA is considered as an important stress hormone in response to environmental stresses and initiating stress responses [38]. It was noticed that GhCAX3 could alleviate ABA sensitivity in tobacco via improving the germination of tobacco seeds, easing the suppression of primary root length and up-regulation of some ABA responsive genes more rapidly and strongly (Figure 3). Compared with S-10 line, lower seed germination rate, more sensitivity in primary root length and impaired response to ABA of *NbDIN* was observed in L-4 line, this might be due to the weaker activity of GhCAX3 which is auto-inhibited by the NRR. According to our research, we assume that GhCAX3 acts as a positive regulator in ABA signal transduction either in germination or seedlings stage. Meanwhile, presence of NRR in GhCAX3 could partially impair the ABA sensitivity in tobacco plants via auto-inhibiting Ca^2+^ transport activity.

Unlike *AtCAX1*, both the full-length and the N-terminal truncated version of *GhCAX3* conferred cold tolerance in yeast as well as in tobacco seedlings. And there was no more difference in cold tolerance between line S-10 and line L-4. Membrane structure is most susceptible to cold stress, being considered to be the primary sites of cold injury [39]. Electrolyte leakage occurs following membrane damage as a typical symptom of cold injury. In our study, S-10 and L-4 lines exhibited less electrolyte leakage, indicating cell membrane was more severely damaged in WT plants compared with that in transgenic lines (Figure 4D). Plants responded to stresses including cold are always associated with the generation of ROS. To protect plants from ROS injury, the activities of antioxidant enzymes are increased, causing a decrease in lipid peroxidation to lessen membrane injury [40]. Consistent with this, increased transcription levels of *NbSOD*, significant elevation of CAT and POD activities were observed in transgenic lines, although the inducible pattern of the key antioxidant enzyme against lipid peroxidation was a little different between S-10 and L-4 plants (Figure 4E-F). This difference of antioxidative systems in function between them might be caused by the constitutive activity of sGhCAX3 in absence of NRR. Except for *NbSOD*, another cold stress responsive marker gene, *NbERD10C*, was also obviously elevated in transgenic plants. It was interesting that the induced abundance of these genes in S-10 plants was a little lower than that in line L-4 (Figure 4G), which was not related to the activity of GhCAX3. Unlike the Ca^2+^ deficiency symptom happened in *sAtCAX1* expressed tobacco and tomato, no Ca^2+^ deficiency induced symptoms was observed in *sGhCAX3* tobacco, which is in a similar manner to *sAtCAX2* expressed plants [41], indicating that GhCAX3 didn’t disturb [Ca^2+^]_cyt_ homeostasis in tobacco cells and was involved in cold stress-induced [Ca^2+^]_cyt_ signal pathway.

This raises an interesting question that why AtCAX1 and GhCAX3, both of which belong to CAX subfamilies, conferred distinct tolerance in seedlings against the same stimuli. Various stimuli could elicit [Ca^2+^]_cyt_ oscillations in spatial-temporal specificity, leading to different signal pathways, and CAXs play an important role during this process [42], [43]. Besides this, Wu et al. speculated that different tissues of rice might use different heat shock triggered Ca^2+^signal transduction mechanisms through their own Ca^2+^ signature due to [Ca^2+^]_cyt_ signature in root cells was distinct from that in epicotyl and leaf cells during heat-shock stress [44]. As *GhCAX3* express predominantly in leaves whereas *AtCAX1* exhibit different expression pattern, they may cause diverse Ca^2+^ signature even under the same stimuli. Thus, the different tolerance of AtCAX1 and GhCAX3 confer plants under cold stimuli might be due to diverse frequencies and amplitudes of [Ca^2+^]_cyt_ oscillations. Another possible reason is probably due to different mechanisms between these two species. Similar observations have been obtained from different CAXs even from same species. It has been observed that *AtCAX1* and *AtCAX4* overexpressed tobacco confer opposite tolerance against the same stimuli under IAA treatment [45].

Although GhCAX3 conferred cold tolerance in tobacco seedlings, entirely different phenotypes against cold stress were obtained from S-10 and L-4 seeds as compared to WT seeds (Figure 4A). This might be caused by different transduction mechanisms responding to low temperature during seed germination and seedling. Besides this, the less sensitivity of L-4 seeds against cold during germination as compared to S-10 seeds should be contributed to the presence of NRR, which partially inhibited the activity of GhCAX3. The observations that genes show different roles during different stages of development are not our unique discovery. Chen et al. also found that the *hy5* mutant of *Arabidopsis* assumed less sensitivity against salt and glucose during germination whereas it was more sensitive against these stresses during seedlings stage [46]. The molecular mechanism of *GhCAX3* in cold induced signaling pathway during different periods of growth should be further investigated.

Though some abiotic stresses such as cold and salt can induce an internal signal transduction in ABA-dependent branch, cold and ABA are thought to be involved in two separate pathways. It has become clear that the signaling networks are interconnected with each other through genes named cross-talk node [34]. To date, several cross-talk nodes mediated cold and ABA pathway have been recognized such as AtSFR6 and AtCIPK3 [47], [48]. Both mutants of these genes in *Arabidopsis* showed not only altered gene induction pattern by cold but also ABA induced gene expression. In our study, we also obtained similar results at molecular level in *GhCAX3* (both full-length and N-terminal truncated form) overexpressing tobacco. The cold responsive gene *NbERD10C* and the ABA response gene *NbDIN* were induced simultaneously by ABA and cold stimuli, indicating that GhCAX3 participated in these two separate pathways. The difference in induced pattern of these two genes under either cold or ABA treatment should be attributed to the NRR. Furthermore, other evidence was observed from germination experiment of *GhCAX3* and *sGhCAX3* seeds during cold stress in the presence of sodium tungstate. The result showed that ABA could alleviate the damage of both *GhCAX3* and *sGhCAX3* seeds on germination impaired by cold, compared with that in WT seeds. These results indicated that GhCAX3 participated in the interaction between ABA and cold signal transduction as a new cross talk node, and the absence of NRR made the germination of transgenic tobacco seeds more sensitive under cold stress in presence of sodium tungstate.

In conclusion, a novel type IA CAX gene, *GhCAX3*, from cotton was identified and characterized. We demonstrated that GhCAX3 possessed Ca^2+^ transport activity which could be auto-inhibited by the NRR. This gene could confer cold tolerance in yeast and tobacco seedlings while enhance tobacco seeds more sensitive to cold stress. Besides this, transgenic tobacco showed impaired ABA sensitivity, indicating GhCAX3 involved in mediating cold and ABA signal transduction. However, the molecular mechanism is still need to be investigated.

## Supporting Information

Figure S1 **Bioinformatics analysis of GhCAX3.** (**A**) Alignment of GhCAX3 with CAXs from other species. The 11 putative transmembrane spans (M1–11) are over lined. The 9-amino acid region (CaD) and the N-Terminal regulatory domain (NRR) are indicated. (**B**) The phylogenetic tree for CAXs by Clustal X software. (**C**) Hydropathy profile of GhCAX3 was predicted according to Anthe analysis, the NRR and acidic motif were indicated. (**D**) The transmembrane location of GhCAX3 predicted by TMMOD (http://molbiol-tools) was indicated by red vertical bars. Blue line and green line indicate inside and outside membrane location, putative transmembrane spans were numbered. CAXs included in alignment are AtCAX1-3(AF461691, AF424628, AF256229) from *Arabidopsis thaliana* and OsCAX1a (BAD06218); OsCAX1b (BAD83660); OsCAX2 (BAD83662); OsCAX3 (BAD83663) from *Oryza sativa*.
(TIF)

Table S1 **Primers used in this study.** Forward and reverses primers indicated by F and R, respectively.
(XLSX)
