# Supplementary figures and images for: GhCAX3 Gene, a Novel Ca2+/H+ Exchanger from Cotton, Confers Regulation of Cold Response and ABA Induced Signal Transduction
Source: PLoS One. 2013 Jun 11;8(6):e66303. doi: 10.1371/journal.pone.0066303 (PMC3679082; doi:10.1371/journal.pone.0066303)

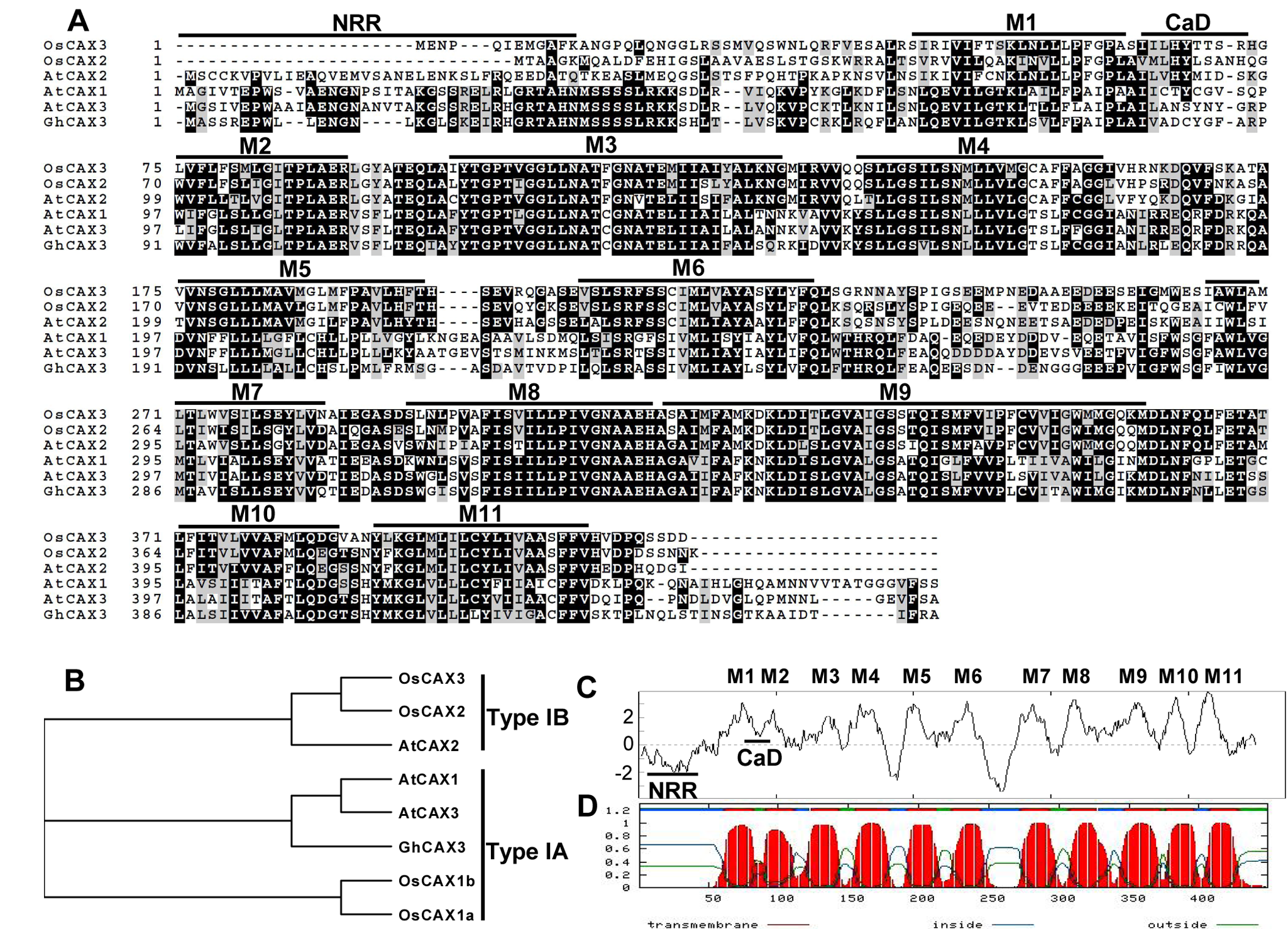

Supplement: Figure S1 — Bioinformatics analysis of GhCAX3. (A) Alignment of GhCAX3 with CAXs from other species. The 11 putative transmembrane spans (M1–11) are over lined. The 9-amino acid region (CaD) and the N-Terminal regulatory domain (NRR) are indicated. (B) The phylogenetic tree for CAXs by Clustal X software. (C) Hydropathy profile of GhCAX3 was predicted according to Anthe analysis, the NRR and acidic motif were indicated. (D) The transmembrane location of GhCAX3 predicted by TMMOD (http://molbiol-tools) was indicated by red vertical bars. Blue line and green line indicate inside and outside membrane location, putative transmembrane spans were numbered. CAXs included in alignment are AtCAX1-3(AF461691, AF424628, AF256229) from Arabidopsis thaliana and OsCAX1a (BAD06218); OsCAX1b (BAD83660); OsCAX2 (BAD83662); OsCAX3 (BAD83663) from Oryza sativa. (TIF) [file pone.0066303.s001.tif]
